# AI-Powered Problem- and Case-based Learning in Medical and Dental Education: A Systematic Review and Meta-analysis

**DOI:** 10.1016/j.identj.2025.100858

**Published:** 2025-06-26

**Authors:** Hongxia Wei, Yuguo Dai, Kaiting Yuan, Kar Yan Li, Kuo Feng Hung, Elaine Mingxin Hu, Angeline Hui Cheng Lee, Jeffrey Wen Wei Chang, Chengfei Zhang, Xin Li

**Affiliations:** a Department of Stomatology, Liuzhou Workers' Hospital, Liuzhou Guangxi, P. R. China; b Division of Restorative Dental Sciences, Faculty of Dentistry, The University of Hong Kong, Hong Kong SAR, China; c Clinical Research Centre, Faculty of Dentistry, The University of Hong Kong, Hong Kong SAR, China; d Division of Applied Oral Sciences and Community Dental Care, Faculty of Dentistry, The University of Hong Kong, Hong Kong SAR, China

**Keywords:** Artificial intelligence, Intelligent tutoring system, Problem-based learning, Case-based learning, Meta-analysis

## Abstract

**Introduction and Aims:**

Advances in artificial intelligence (AI) technology have generated a revolution in medical and dental education, which may offer promising solutions to tackle the challenges of traditional problem-based learning (PBL) and case-based learning (CBL). The objective of this study was to assess the available evidence concerning AI-powered PBL/CBL on students’ knowledge acquisition, clinical reasoning capability and satisfaction.

**Methods:**

An electronic search was carried out on PubMed, MEDLINE, the Cochrane Central Register of Controlled Trials and Web of Science. Clinical trials published in English with full text available, which implemented AI technologies in PBL/CBL in the medical/dental field and evaluated knowledge acquisition, clinical reasoning and/or satisfaction were included. The quality assessment was conducted using RoB 2 by two calibrated assessors. Data synthesis and meta-analysis were performed, the standardised mean difference (SMD) or standardised mean (SM) and 95% confidence intervals (CIs) were calculated, and heterogeneity was quantified.

**Results:**

Six randomized controlled trials were included, with an overall risk of bias judged to have ‘some concerns’. For knowledge acquisition, 4 studies were included in the meta-analysis. A low heterogeneity (I² = 20%) was detected and a fixed-effect model was utilised. Compared with the control group, the AI intervention significantly improved knowledge acquisition by 46% (95% Cls [0.18-0.73], *P* = .001). For clinical reasoning capability, due to methodological and measurement heterogeneity among studies, statistical analysis was not feasible. Three studies were selected for the meta-analysis of students’ satisfaction. Heterogeneity was moderate (I² = 32%), and a generic inverse variance method was selected. The pooled SM score was 0.7 (95% Cls [0.47-0.92]), and the overall effect was statistically significant (*P* < .00001).

**Conclusion:**

Despite limitations such as the limited number of included studies and the overall risk of bias concerns, AI-powered PBL/CBL has the potential to enhance students’ knowledge acquisition and learner satisfaction compared to traditional learning approaches.

**Clinical Relevance:**

Not applicable.

## Introduction

Problem-based learning (PBL) and case-based learning (CBL) are student-centred, inquiry-based pedagogical approaches which are commonly used in medical and dental education.^1^^,^^2^ Compared with traditional passive teaching approaches, students are more actively engaged in PBL and CBL, where they work collaboratively in small groups to solve clinical problems based on real-life scenarios.^3^^,^^4^ Previous studies showed that PBL and CBL facilitated the development of critical thinking skill, clinical reasoning capability, analytic and problem-solving skill, as well as self-directed learning ability.^5^, ^6^, ^7^, ^8^ Systematic reviews and meta-analyses showed that PBL and CBL significantly enhanced students’ knowledge acquisition as compared to traditional education methods.^9^^,^^10^

In spite of these benefits, the limitations and challenges of PBL and CBL including high workload and delivery costs, the requirement of special training for facilitators, the lack of standardized clinical cases, increased demands for case materials, hardware, software and other facilities^11^, ^12^, ^13^ may hinder their application in managing a large cohort of students.

In recent years, rapid advancements in artificial intelligence (AI) technology have generated a revolution in healthcare education,^14^, ^15^, ^16^ which may offer promising solutions to tackle these challenges. Large language models (LLMs) such as ChatGPT (OpenAI), which can automatically process and generate natural language, have been integrated into PBL/CBL to enrich the learning experience.^17^^,^^18^ Intelligent tutoring systems (ITSs) such as COMET (Collaborative MEdical Tutor) and METEOR (Medical Tutor Employing Ontology for Robustness) are computer-based educational platforms which utilise AI and adaptive technologies to mimic human tutoring by providing real-time, customised and interactive feedback to learners.^19^^,^^20^ AI can further assist PBL and CBL by leveraging generative models such as LLMs, generative adversarial networks (GANs), variational autoencoders (VAEs) and diffusion models to create synthetic multimodal cases which integrate medical text, imaging and physiological signals to stimulate realistic clinical scenarios.^21^^,^^22^ In addition, the integration of virtual reality (VR), augmented reality (AR) and haptic systems has revolutionised the development of virtual patients, enabling learners to practise communication, clinical diagnosis, decision-making and treatment in a safe and adaptative environment.^23^^,^^24^

Despite the growing interest and popularity of AI-powered medical and dental education, there is a lack of a comprehensive evaluation of its effectiveness in improving the learning outcomes of PBL and CBL. Therefore, in this systematic review and meta-analysis, we aim to critically assess the available evidence on students’ knowledge acquisition, clinical reasoning capability and satisfaction. The overall objective is to provide deeper insights into this emerging education trend, as well as to offer valuable recommendations for future development and optimisation of AI-powered educational tools and platforms.

## Method

### Protocol registration and review question

This systematic review was conducted following the PRISMA 2020 statement, and the protocol has been registered in the PROSPERO database (CRD42024626780). The research question was formulated using the PICO framework: For medical and dental students [Population (P)], does AI-powered PBL or CBL [Intervention (I)] improve knowledge acquisition, clinical reasoning capability and satisfaction [Outcome (O)], compared to PBL/CBL without the use of AI technologies or traditional lectures/seminars [Comparison (C)]?

### Information sources and search strategy

The search was carried out in four databases including PubMed, MEDLINE, the Cochrane Central Register of Controlled Trials (CENTRAL) and Web of Science. To capture grey literature, a supplementary search was performed in Google Scholar, targeting unpublished manuscripts, conference papers, and research reports. The detailed search strategy is illustrated in Table 1. The first search was conducted on 11 November 2024. An update was performed on 24 March 2025.

**Table 1:** The detailed search strategy for each database.

Database	Searching strategy
PubMed	((((((problem-based learning[Title/Abstract]) OR (problem-oriented learning[Title/Abstract])) OR (flipped classroom[Title/Abstract])) OR (inquiry-based learning[Title/Abstract])) OR (case-based learning[Title/Abstract])) AND (((((artificial intelligence[Title/Abstract]) OR (deep learning[Title/Abstract])) OR (machine learning[Title/Abstract])) OR (data science[Title/Abstract])) OR (intelligent systems[Title/Abstract]))) AND (((((dentistry[Title/Abstract]) OR (medicine[Title/Abstract])) OR (healthcare[Title/Abstract])) OR (medical[Title/Abstract])) OR (dental[Title/Abstract]))
MEDLINE	(artificial intelligence or deep learning or machine learning or data science or intelligent systems).mp. [mp=title, book title, abstract, original title, name of substance word, subject heading word, floating sub-heading word, keyword heading word, organism supplementary concept word, protocol supplementary concept word, rare disease supplementary concept word, unique identifier, synonyms, population supplementary concept word, anatomy supplementary concept word] AND (problem-based learning or problem-oriented learning or flipped classroom or inquiry-based learning).mp. [mp=title, book title, abstract, original title, name of substance word, subject heading word, floating sub-heading word, keyword heading word, organism supplementary concept word, protocol supplementary concept word, rare disease supplementary concept word, unique identifier, synonyms, population supplementary concept word, anatomy supplementary concept word] AND (dentistry or medicine or healthcare or medical or dental).mp. [mp=title, book title, abstract, original title, name of substance word, subject heading word, floating sub-heading word, keyword heading word, organism supplementary concept word, protocol supplementary concept word, rare disease supplementary concept word, unique identifier, synonyms, population supplementary concept word, anatomy supplementary concept word]
CENTRAL	((problem-based learning):ti,ab,kw OR (problem-oriented learning):ti,ab,kw OR (flipped classroom):ti,ab,kw OR (inquiry-based learning):ti,ab,kw OR (case-based learning):ti,ab,kw) AND ((artificial intelligence):ti,ab,kw OR (deep learning):ti,ab,kw OR (machine learning):ti,ab,kw OR (data science):ti,ab,kw OR (intelligent systems):ti,ab,kw) AND ((dentistry):ti,ab,kw OR (healthcare):ti,ab,kw OR (medicine):ti,ab,kw OR (medical):ti,ab,kw OR (dental):ti,ab,kw)
Web of Science	(((((TS=(problem-based learning)) OR TS=(problem-oriented learning)) OR TS=(flipped classroom)) OR TS=(inquiry-based learning)) OR TS=(case-based learning)) AND (((((TS=(artificial intelligence)) OR TS=(deep learning)) OR TS=(machine learning)) OR TS=(data science)) OR TS=(intelligent systems)) AND (((((TS=(dentistry)) OR TS=(medicine)) OR TS=(healthcare)) OR TS=(medical)) OR TS=(dental))

### Study selection

Data from four databases were combined and duplicates were removed. The inclusion and exclusion criteria are listed in Table 2. Clinical trials published in English with full text available which implemented AI technologies in PBL/CBL in medical or dental education and evaluated students’ knowledge acquisition, clinical reasoning capability and/or satisfaction were included. The exclusion criteria were irrelevant topics, review papers, comments, editorial or short communications, non-English, lacking an appropriate control group and unavailable full texts or missing data. From included studies, data including study design (type, duration, sample size), participant demographics (age, gender, academic year), intervention measures (AI intervention type, name of the AI tools, PBL/CBL delivery) and outcome metrics (knowledge acquisition, clinical reasoning capability, satisfaction) were extracted. The selection and data extraction were conducted independently by 2 calibrated investigators. Discrepancies/ disagreements were resolved through consensus-based discussion. If an agreement could not be reached, a third researcher was consulted.

**Table 2:** Inclusion and exclusion criteria.

Parameter	Inclusion criteria	Exclusion criteria
Population (P)	Medical or dental students who were participating in PBL/CBL	Non-medical or dental students Students who were not engaged in PBL/CBL
Intervention (I)	AI technologies were applied in PBL/CBL	
	AI-assisted teaching software, such as intelligent tutoring systems	
	Platforms that use AI for case analysis and problem-solving	
	Introducing AI tools to assist problem analysis in group discussions	
Comparison (C)	Traditional teaching methods PBL/CBL without the use of AI technologies	No appropriate control group
Outcome (O)	Students' knowledge acquisition, clinical reasoning capability, satisfaction	
Study design	Clinical trials	Review, comment, editorial, short communication
Language	English	Other than English

### Quality assessment and data synthesis

The risk of bias of each included study was assessed independently by 2 investigators using version 2 of the Cochrane risk-of-bias tool for randomized trials (RoB 2). Bias arising from 5 domains including the randomisation process (D1), deviations from intended interventions (D2), missing outcome data (D3), outcome measurement (D4) and selection of the reported result (D5) was evaluated. The risk of bias for each domain was judged as ‘low risk of bias’, ‘some concerns’, or ‘high risk of bias’. When all domains were judged to be at low risk of bias, the overall risk-of-bias judgement was ‘low risk of bias’; when some concerns were raised in 1 or more domains, the overall risk-of-bias judgement was ‘some concerns’; the overall risk-of-bias was judged to be at ‘high risk of bias’ when at least 1 domain showed a high risk of bias or some concerns were raised in multiple domains which substantially lowered the confidence in the result. In the case of disagreement, differences were discussed until a consensus was reached.

A descriptive summary of the included studies was initially prepared to assess the quantity, characteristics and variability of the data. Meta-analysis was performed using Review Manager (RevMan) version 5.4 when studies were sufficiently homogeneous in terms of assessment tools and interventions and when data approximated a normal distribution. Primary outcomes were knowledge acquisition and clinical reasoning capability, and the effect measure was standardised mean differences (SMD). Secondary outcome was students’ satisfaction; standardised means (SM) without comparison was selected as an effective measure. Both effect measures were extracted only when the normal distribution assumptions were met. When studies used only non-parametric test and summary statistics, such as medians with standard deviations or medians with interquartile ranges (IQRs), skewness was first estimated from reported summaries using validated methods.^25^ If the studies passed the skewness test, means and standard deviations (SDs) were estimated from medians and IQRs (or ranges) using established transformation methods^26^, ^27^, ^28^ to enable the calculation of effect measures for meta-analysis. Studies with severely skewed data (which did not pass the skewness test) were excluded from quantitative synthesis but summarised descriptively, accompanied by *P* values from non-parametric tests (e.g. Mann-Whitney U). For the effect measures, the corresponding 95% confidence intervals (CIs) were also estimated. Heterogeneity was quantified by I² statistics (I² < 25%: low; 25%-50%: moderate; >75%: high),^29^ and publication bias was assessed using funnel plots, Begg’s rank test or Egger's regression test (≥10 studies). Continuous data were pooled using fixed-effect models (I² < 50%) or random-effects models (I² ≥ 50%) with subgroup analyses according to control type (PBL/CBL without AI integration or traditional lectures/seminars). In cases of high heterogeneity, a sensitivity analysis was performed to evaluate the impact of each study on the pooled results. When statistical pooling was not feasible due to heterogeneity or insufficient data, results were summarised using descriptive statistics.

## Results

### Search results

**Fig. 1:**
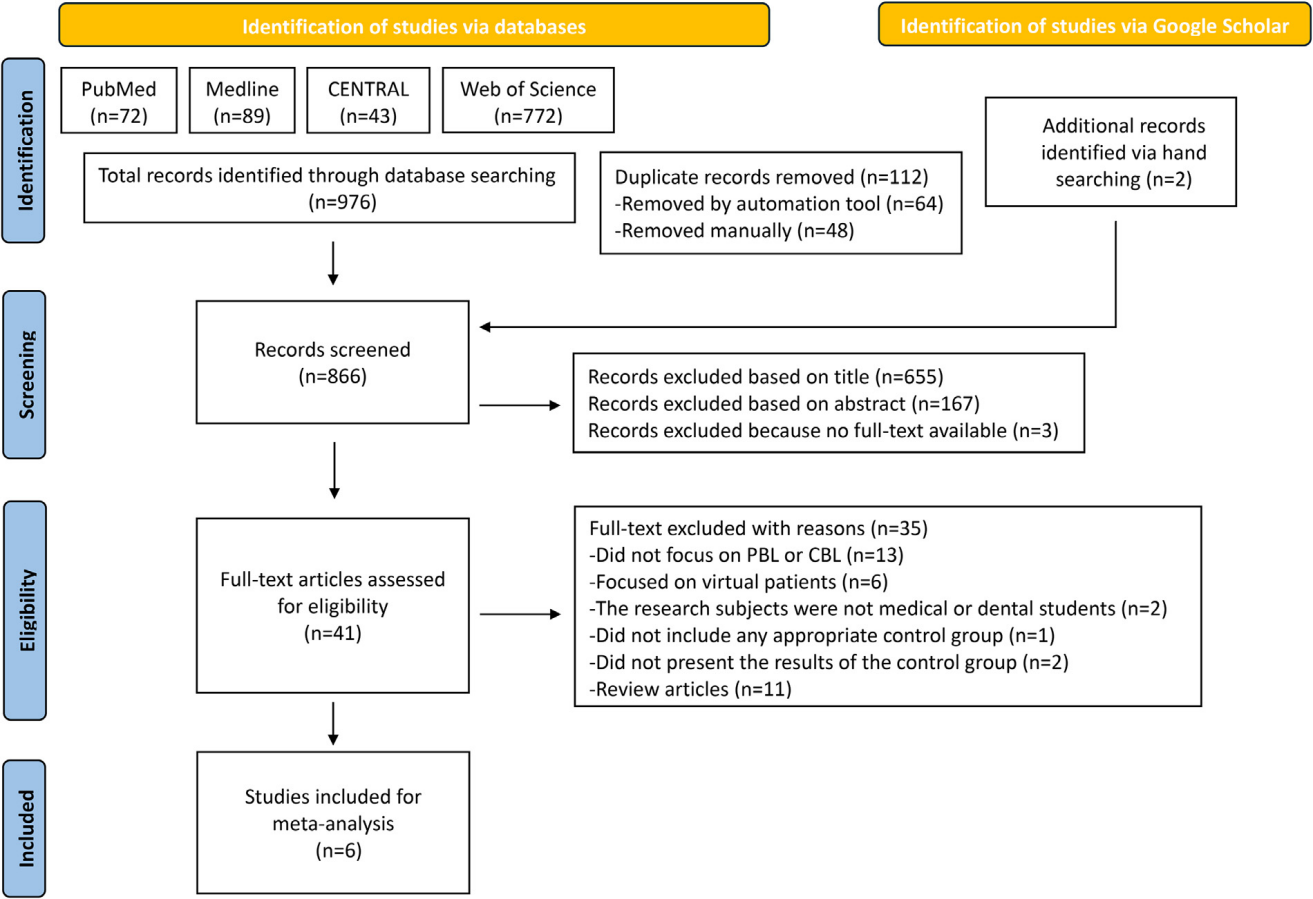
A PRISMA flowchart illustrating the article screening process.

The selection process and the search results are outlined in the PRISMA flow diagram in Figure 1. Forty-one full-text articles were assessed for eligibility, and 35 of them were excluded. The reasons for exclusion were as follows: 13 articles did not focus on PBL/CBL, 6 articles investigated virtual patients, the research subjects of 2 articles were not students, 1 article did not include any appropriate control group, 2 articles did not present the results of the control, and 11 articles were reviews. Finally, 6 articles that met the inclusion criteria were included,^18^^,^^30^, ^31^, ^32^, ^33^, ^34^ and they were all randomised controlled trials (RCTs). The details of the included studies are summarised in Table 3.

**Table 3:** General characteristics of the included studies.

Author (year)	Journal	Population	Learning approach	Intervention	Comparison	Outcome		
						Parameter	Tool	Findings
Suebnukarn (2009)	Journal of Dental Education	36 second-year dental students (*n* = 18 per group)	PBL	ITS COMET	Traditional PBL	Clinical reasoning capability	CRP approach	Clinical reasoning gains for COMET-tutored students were similar to those for human-tutored students, the average post-test score for COMET students (61.45) was comparable to that obtained for human-tutored students (59.46) (Mann-Whitney, *P* = .058).
Aparicio et al. (2012)	Computers & Education	60 second-year medical students (*n* = 34 for AI group and *n* = 26 for the control)	CBL	IIAS	Free internet-based learning in CBL	Knowledge acquisition and satisfaction	Objective tests and subjective questionnaires	(1) Knowledge acquisition: the results of the objective test in IIAS group was not significantly different from free internet-based learning group, the overall percentage of correct answers was 78.53% and 76.92%, respectively. (2) Satisfaction: the results of subjective questionnaires showed that 58.82% and 64.71% of the students strongly agreed or agreed about the usefulness of the system for finding relevant clinical case terms and for reducing the time taken to understand the text, respectively; 61.76% felt that the interface was user-friendly, 67.65% considered the required information was easy to find and felt comfortable using the system, 76.47% perceived the system speed reasonable and 94.12% found the system easy to use.
Wu et al. (2020)	Annals of Translational Medicine	38 second-year medical students (*n* = 19 per group)	PBL	ITS CC-Crusier	Traditional lectures	Knowledge acquisition and satisfaction	Test covering the signs, diagnosis and treatment, as well as a questionnaire	(1) Knowledge acquisition: the post-lecture scores were significantly higher than the pre-lecture scores for both groups (*P* < .001), the total post-lecture score of AI tutoring was significantly higher than that of traditional lectures (*P* = .034). (2) Satisfaction: all students in AI tutoring group were satisfied and agreed that the platform was helpful, effective, and beneficial; a higher requirement of hardware and more time needed for previewing were reported.
Zhao et al. (2023)	International Journal of Medical Informatics	72 medical undergraduates (*n* = 36 per group)	CBL	WFO	Traditional CBL	Knowledge acquisition and satisfaction	Examination and teaching assessment questionnaire	(1) Knowledge acquisition: the exam score in WFO-based group (89.50 ± 5.86) was similar as that of the control group (85.92 ± 8.33) (*P* = .139), the pass rate was 100% for both groups (≥60). (2) Satisfaction: the overall satisfaction of WFO group was higher than the control group (89.25 ± 5.92 versus 80.75 ± 3.42, *P* = .001), the difference was mainly reflected in cultivating independent learning ability, increasing knowledge mastery, enhancing learning interest, and increasing course participation, no significant difference was observed in improving the ability of expression.
Wang et al. (2024)	Medical Teacher	101 medical undergraduates and postgraduates (*n* = 51 for AI group and *n* = 50 for control group)	PBL	LearnGuide, a customized tool based on ChatGPT	Traditional PBL	Clinical reasoning capability	CCTT Level Z	Both groups showed higher CCTT scores after 12 weeks compared to baseline; the difference between the 2 groups was not statistically significant at 6 weeks, while by 12 weeks, the Learn Guide group showed a significant improvement (*P* < .001), which remained significant at 14-week follow-up (*P* < .001).
Zeng et al. (2025)	BMC Medical Education	42 fifth-year medical interns (*n* = 21 per group)	PBL	ChatGPT	Traditional teaching	Knowledge acquisition, clinical reasoning capability, and satisfaction	Theoretical knowledge exam, mini-CEX assessment and questionnaire	(1) Knowledge acquisition: the scores of theoretical knowledge exam in ChatGPT-assisted group were significantly higher than those in traditional teaching group 3 days after the course ended (93.90 ± 3.65 versus 90.33 ± 4.08; *P* < .01). (2) Clinical reasoning capability: mini-CEX evaluation showed that the ChatGPT group demonstrated statistically significant improvements in clinical judgment (*P* < .01) and overall clinical competence (*P* < .01) compared to the control group. (3) Satisfaction: the students gave highly positive feedback on ChatGPT teaching method and no dissatisfied situation was reported.

CCTT, Cornell Critical Thinking Test; COMET, COllaborative MEdical Tutor; CRP, clinical reasoning problem; IIAS, intelligent information access system; ITS, intelligent tutoring system; Mini-CEX, Mini Clinical Evaluation Exercise; WFO, Watson for Oncology.

### Quality assessment of included studies

**Fig. 2:**
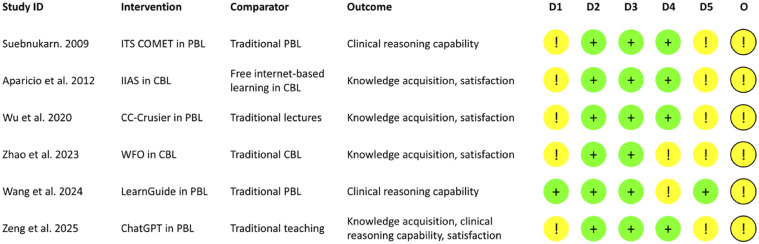
The risk of bias in each study included in this systematic review. D1: bias arising from the randomization process; D2: bias due to deviations from intended interventions; D3: bias due to missing outcome data; D4: bias in measurement of the outcome; D5: bias in selection of the reported result. The green, yellow, and red colours represent a low risk of bias, some concerns, and a high risk of bias, respectively. CBL, case-based learning; COMET, COllaborative MEdical Tutor; IIAS, intelligent information access system; ITS, intelligent tutoring system; PBL, problem-based learning; WFO, Watson for Oncology.

The overall risk of bias for all studies was judged with ‘some concerns’ (Figure 2). For the randomisation process (D1), 5 studies mentioning random sampling without elaborating on the randomization process^18^^,^^30^, ^31^, ^32^, ^33^ were categorized as ‘some concerns’. For deviations from intended interventions (D2), participants from all studies adhered to assigned intervention; only in 1 study a student was lost to follow-up and 1 withdrew,^34^ suggesting ‘low risk of bias’. D3 missing outcome data was judged as ‘low risk of bias’ because data were available for all studies. As for the measurement of the outcome (D4), knowledge acquisition was assessed using objective multiple-choice tests^31^ or theoretical knowledge exams,^18^^,^^32^ and clinical reasoning capability was evaluated using clinical reasoning problem (CRP) approach,^30^ representing ‘low risk of bias’. In one study, knowledge acquisition was measured using the same examination pre- and post-intervention,^33^ so the potential testing effect could have resulted in an overestimation of the outcome. To evaluate clinical reasoning capability, one study used the Cornell Critical Thinking Test (CCTT) Level Z which relied on a subjective self-assessment,^34^ and these participants were aware of group allocation, which might have resulted in response bias. Therefore, for the latter 2 studies, a judgement of ‘some concerns’ was indicated.^33^^,^^34^ Only 1 study was preregistered, and the outcomes reported were in accordance with those specified in preregistered protocol,^34^ indicating ‘low risk of bias’ in selection of the reported result (D5), while the other 5 studies were identified as ‘some concerns’. Due to the limited number of studies included, publication bias could not be assessed.

### Knowledge acquisition

**Fig. 3:**
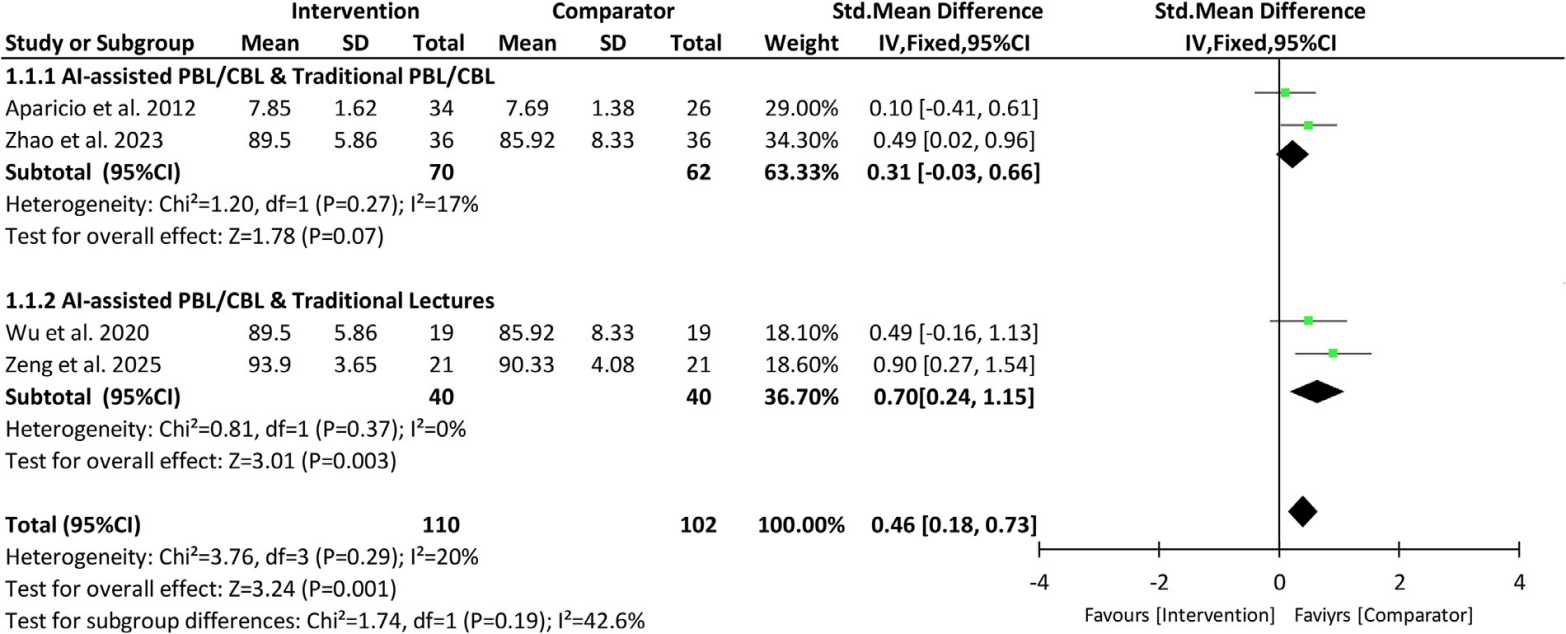
A forest plot of meta-analysis of the studies comparing students’ knowledge acquisition in AI-powered PBL/CBL with PBL/CBL without AI integration or traditional lectures. The box represents the point estimate of the SMD of the study, the horizontal line indicates the 95% CI, and the size of the box reflects the weight of the study in relation to the pooled estimate. The diamond represents the overall effect estimate derived from the meta-analysis.

Four studies using objective examinations for knowledge acquisition were selected for meta-analysis,^18^^,^^31^, ^32^, ^33^ and subgroup analyses were performed based on different types of control groups (PBL/CBL without AI integration or traditional lectures/seminars) (Figure 3).

A total number of 110 and 102 students were included in the meta-analysis of the AI-assisted group and the control group, respectively. A low heterogeneity was observed among the 4 included studies (*P* = .29 and I² = 20%), and a fixed-effect model was selected. The overall result showed that AI intervention significantly improved students’ knowledge acquisition by 46% when compared to the control group (95% Cls [0.18-0.73], *P* = .001). No statistical difference was detected between the 2 subgroups (*P* = .19). Subgroup analysis of the AI intervention group and the traditional PBL/CBL group^31^^,^^33^ indicated that the knowledge acquisition in the AI group was 31% higher than those in the traditional group, but this difference was not statistically significant (95% Cls [-0.33 to 0.66], *P* = .07). Subgroup analysis of the AI intervention group and the traditional lectures group^18^^,^^32^ showed that the knowledge acquisition in the AI group was 70% higher than that in the traditional lectures group, and the difference was statistically significant (95% Cls [0.24-1.15], *P* = .003).

### Clinical reasoning capability

**Fig. 4:**
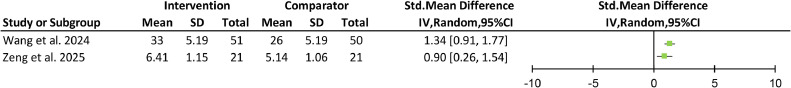
A forest plot of the studies comparing clinical reasoning capability of AI-powered PBL/CBL with PBL/CBL without AI integration or traditional lectures. The box represents the point estimate of the SMD of the study, the horizontal line indicates the 95% CI, and the size of the box reflects the weight of the study in relation to the pooled estimate.

The impact of AI-powered PBL/CBL on clinical reasoning capability was investigated in 3 studies, each employing distinct assessment approaches including CRP, CCTT Level Z and mini Clinical Evaluation Exercise (mini-CEX).^18^^,^^30^^,^^34^ Methodological heterogeneity arose from the use of diverse instruments that evaluated different constructs within the domain of clinical reasoning. Measurement heterogeneity further limited data synthesis because sufficient data for SMD calculation were provided in only 2 studies,^18^^,^^34^ whereas in the other study,^30^ non-parametric testing was employed without reporting the necessary summary statistics for normality assessment, thus precluding effect size estimation. Therefore, statistical pooling was not feasible, and a descriptive synthesis was employed to summarise the findings in Figure 4. Previous studies using the CRP approach showed that the clinical reasoning gains for AI-tutored students were similar to those for human-tutored students, with no significant difference in the average post-test score observed for the 2 groups (Mann-Whitney, *P* = .058).^30^ This study used non-parametric tests for comparison, and the normality test was not fulfilled.^30^ Compared with traditional PBL, the integration of LearnGuide, a customised tool based on ChatGPT, showed higher effectiveness in enhancing clinical reasoning capability (SMD = 1.34, 95% CIs [0.91,1.77]).^34^ A weaker favourable effect was observed in ChatGPT-assisted PBL when compared to traditional teaching (SMD = 0.90, 95% CIs [0.26,1.54]).^18^

### Satisfaction with AI-powered PBL/CBL approach

**Fig. 5:**
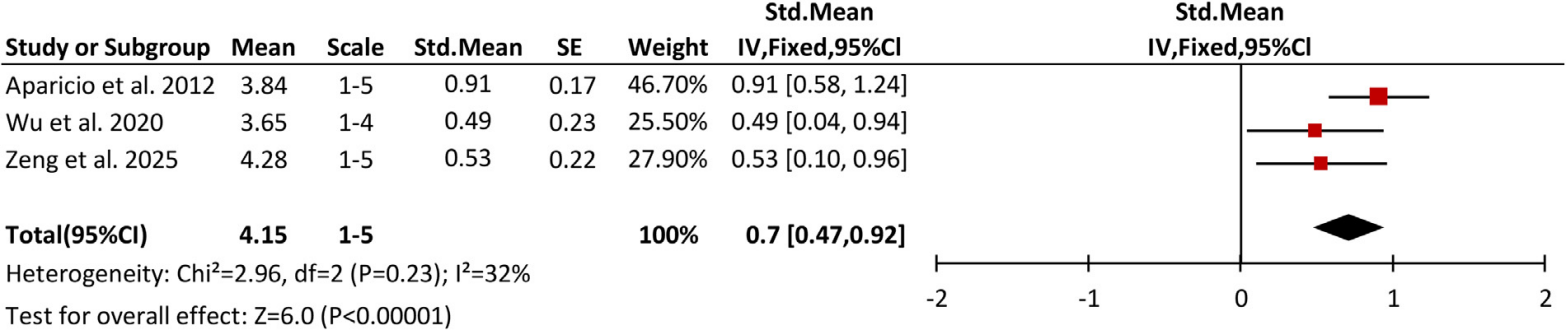
A forest plot of a meta-analysis of the studies comparing students’ satisfaction with AI-powered PBL/CBL. The box represents the point estimate of the SM of the study, the horizontal line indicates the 95% CI, and the size of the box reflects the weight of the study in relation to the pooled estimate. The diamond represents the overall effect estimate derived from the meta-analysis.

Students’ satisfaction with the AI-assisted PBL/CBL approach was assessed in 4 studies^18^^,^^31^, ^32^, ^33^ using subjective questionnaires. The questionnaires were distributed to students from both the AI intervention group and the control group in 1 study.^33^ For the other 3 studies,^18^^,^^31^^,^^32^ the questionnaires were answered only by students in the AI group, and these studies with 74 participants in total were included in the meta-analysis (Figure 5).

Given that the answers to the questionnaires regarding satisfaction were rated on different scales ranging from 1 (strongly disagree) to 4 or 5 (strongly agree), SM was selected as the effect measure. The result of each question was first converted to the average satisfaction score and SD individually, and then the average satisfaction score and the corresponding SD for all questions included were calculated, after which the SM and the corresponding standard error (SE) were estimated. Heterogeneity was moderate (I² = 32%), and a fixed-effect generic inverse variance method was selected for meta-analysis. The pooled SM satisfaction score was 0.7 (95% Cls [0.47-0.92]), indicating students were generally satisfied with AI-assisted PBL/CBL, and the variability in the satisfaction score across 3 studies was moderate. The overall effect was statistically significant (Z = 6, *P* < 0.0001), suggesting that the observed high satisfaction with AI-assisted PBL/CBL was consistent and robust across all included studies.

## Discussion

This systematic review and meta-analysis of 6 RCTs showed that compared with PBL/CBL without AI integration, traditional lectures or seminars, AI-empowered PBL/CBL facilitated an intensive interaction between students and the system and significantly improved students’ knowledge acquisition. Moreover, high satisfaction with this emerging educational modality was observed. The findings were in line with previous studies^35^, ^36^, ^37^ which highlighted the potential of AI technology to revolutionise medical and dental education by creating personalised learning experiences and improving students’ engagement.

Regarding the application scenarios of AI technology in PBL/CBL, 4 out of the 6 included studies used ITSs including COMET,^30^ CC-Crusier,^32^ LearnGuide^34^ and ChatGPT.^18^ COMET was developed in 2006 by Dr. Suebnukarn from Thammasat University Dental School in Thailand.^30^ In this system, preclinical domain knowledge of each problem scenario was incorporated and a multimodal interface consisting of text and graphics was presented. Students were asked to create a network of hypotheses and links, which were subsequently verified by experts in the field. Based on Bayesian networks and generic tutoring algorithms, COMET could generate tutoring hints. The generated hints and student dialogues were displayed in a discussion pane to enable an open discussion among students.^30^ An earlier study showed no significant difference between the instructions generated by COMET and those of experienced human tutors, indicating a high degree of agreement.^19^ In 2009, COMET-integrated PBL was introduced to second-year dental students, and the clinical reasoning gains obtained from this COMET system in the field of stroke and heart attack were similar as those obtained from human-tutored PBL.^30^ In 2013, the next version of the COMET system METEOR was developed, which used the Unified Medical Language System (UMLS) Semantic Network as the domain ontology.^38^ Different from COMET, which created the problem scenarios by encoding the expert solutions manually into the system, METEOR combined problem solutions collected from experts with UMLS tables to form a broader domain model. The development time of a problem scenario was largely reduced from 1 month for 1 person in the COMET system to 4-5 hours for 1 person. Unfortunately, the previous study evaluated the clinical reasoning capability of only those students following the METEOR session.^38^ With the absence of a control group, it is challenging to draw conclusions on the effectiveness of this system.

CC-Cruiser, an AI platform based on image features for the diagnosis and evaluation of paediatric cataracts, was incorporated in PBL.^32^ Compared to traditional lectures, CC-Cruiser was more effective in conveying knowledge on signs and diagnoses (*P* = .016), while for treatment strategy, no significant difference was detected.^32^ LearnGuide, a customised version of ChatGPT, was designed to support PBL in medical education.^34^ LearnGuide intervention was claimed to effectively improve students’ critical thinking skills and self-directed learning abilities, and these benefits appeared more pronounced over 12 weeks of training.^34^ However, it should be noted that in this study, solely subjective self-assessments were selected to measure the learning outcomes, and the potential influence of self-report bias could not be ruled out. In addition, ChatGPT was also incorporated into PBL by generating open-ended questions, as well as creating simulated patients and interviews, which enriched students’ learning experience by allowing the practice of history-taking.^18^ The incorporation of ChatGPT resulted in a significant improvement in students’ medical interviewing skills, clinical judgement and overall clinical competence compared to traditional learning.^18^

Apart from ITSs, an intelligent information access system using Medlineplus (National Institutes of Health) and Freebase (Google) as ontologies, was developed.^31^ This system generated students’ knowledge acquisition similar to free internet search, but the drawbacks such as the slow annotation process of the system and the presence of irrelevant information were reported, which may result in a lower satisfaction level with the system (3.84 out of 5) compared with CC-Cruiser (3.65 out of 4) and ChatGPT (4.28 out of 5). AI-generated decision-making system WFO (Watson for Oncology, International Business Machines) was recently introduced to CBL in clinical oncology, and similar knowledge acquisition with a higher overall satisfaction score was observed in WFO-integrated CBL compared to traditional CBL.^33^

Although the available data suggest that these AI-empowered systems/platforms may be beneficial for enhancing students’ knowledge acquisition, the limitations such as the additional time and effort needed for building problem/case models,^30^ the high requirement of hardware^32^ and the risk of students’ over-reliance^39^^,^^40^ should be addressed in future studies. Moreover, the knowledge base of problem/case scenarios should be updated in a timely manner^41^ so that the students can be exposed to the most recent knowledge and concepts, which will further optimise their learning experience. Finally but no less importantly, the roles of facilitators remain crucial and indispensable. During the learning process, tutors/instructors not only educated students on the utilization of an AI-powered system/platform but also verified the accuracy of the information provided by AI and provided personalized feedback.^18^^,^^30^^,^^32^ To realise the full potential of this innovative approach, AI technology should be combined with skilled facilitators.

The limitations of this systematic review and meta-analysis should be considered and the results should be interpreted with caution. First, the exclusion of non–English-language studies may have introduced language bias and reduced the representativeness of the results. Second, the number of included studies was relatively small, which may limit the reliability and generalisability of the finding. Due to the limited number of studies included in meta-analyses, a robust assessment of publication bias was not feasible. Additionally, the absence of a control group precluded the evaluation of students' satisfaction using SMD. Instead, satisfaction levels could only be reported as SM in terms of standard deviations, limiting the comparability of the findings. It should also be noted that the overall risk of bias for all included studies was judged to have ‘some concerns’. Furthermore, there is insufficient evidence supporting the long-term effectiveness of AI-powered learning. Of the 6 included studies, 4 evaluated the learning outcomes immediately after a single learning session,^18^^,^^30^, ^31^, ^32^ which might not be sufficient to fully assess the development of complex skills such as critical thinking, clinical reasoning, problem-solving and self-directed learning, regarded as the main benefits of PBL and CBL. The results of objective examinations obtained from the included studies might not accurately reflect the development of students’ complex skills. Additionally, students might have responded more positively because of the novelty of the AI-integrated approach, which could have contributed to the high level of satisfaction and might not reflect their sustained engagement and satisfaction over time.

To address these limitations, additional high-quality and longitudinal RCTs with large numbers of students with varying levels of prior knowledge and experience and at different educational stages are highly desirable. A variety of comprehensive objective exams as well as subjective assessments such as essays, case studies or reflective journals should be used to provide a more comprehensive understanding of the effectiveness and sustainability of AI-integrated learning.

## Conclusions

The integration of novel AI technology in PBL and CBL may enhance students’ knowledge acquisition and satisfaction, which holds promise for generating a positive paradigm shift in medical and dental education.

## Conflict of interest

None disclosed.
